# Recent Advances: Decoding Alzheimer’s Disease With Stem Cells

**DOI:** 10.3389/fnagi.2018.00077

**Published:** 2018-03-22

**Authors:** Yi Fang, Ting Gao, Baorong Zhang, Jiali Pu

**Affiliations:** Department of Neurology, The Second Affiliated Hospital, Zhejiang University School of Medicine, Hangzhou, China

**Keywords:** Alzheimer’s disease, neurogenesis, stem cell transplantation, induced pluripotent stem cell, disease modeling

## Abstract

Alzheimer’s disease (AD) is an irreversible neurodegenerative disorder that destroys cognitive functions. Recently, a number of high-profile clinical trials based on the amyloid cascade hypothesis have encountered disappointing results. The failure of these trials indicates the necessity for novel therapeutic strategies and disease models. In this review, we will describe how recent advances in stem cell technology have shed light on a novel treatment strategy and revolutionized the mechanistic investigation of AD pathogenesis. Current advances in promoting endogenous neurogenesis and transplanting exogenous stem cells from both bench research and clinical translation perspectives will be thoroughly summarized. In addition, reprogramming technology-based disease modeling, which has shown improved efficacy in recapitulating pathological features in human patients, will be discussed.

## Introduction

Alzheimer’s disease (AD) is a chronic neurodegenerative disorder characterized by progressive cognitive decline. AD affects 5–7% of older adults globally (Prince et al., 2013), and the expected number of affected patients is expected to grow continuously as the population ages in most countries. Currently, however, there is no cure for this condition. The Food and Drug Administration approved and actively marketed drugs for AD, including cholinesterase inhibitors and *N*-Methyl-D-Aspartate antagonists, whose effects improve daily functions to a certain degree (Rogers and Friedhoff, 1996; Tariot et al., 2004), yet they are not capable of altering disease progression. Tremendous efforts have been made to develop novel therapeutics to potentially reverse disease progression. Among the ongoing clinical trials designed to modify AD, a majority of them are intended to ameliorate Aβ, including β-secretase inhibitors, immunotherapies, and anti-aggregation agents (Cummings et al., 2017). Recently, several pioneering spotlighted trials targeting Aβ have met with dissatisfying results in terms of improved cognitive function (Doody et al., 2013; Salloway et al., 2014). One cannot jump to the conclusion that these negative clinical outcomes refute the prevailing amyloid cascade hypothesis, yet lessons should be learned from these dissatisfying results. Interestingly, successful elimination of amyloid in animal models, which typically overexpress *APP* or presenilin (*PS1*, *PS2*) genes, does not guarantee successful cognitive restoration in human patients. On the one hand, AD is a complex disease involving multiple cell types and cellular processes; therefore, targets other than amyloid should be considered and tested. On the other hand, developing solid models that better mimic disease pathologies in terms of NFTs, neuronal loss, and cellular interactions will undoubtedly benefit drug screening and mechanistic investigations. In this review, we will discuss how current advances in stem cell technology might address these unmet needs.

## Efforts to Promote Endogenous Neurogenesis

Substantial neuronal loss is observed even in mild AD patients (Gómez-Isla et al., 1996). Intuitively, increasing the number of neurons or replacing lost neurons are potential therapeutic strategies for AD. Stem cells are capable of renewing themselves continuously and differentiating into specialized cells, including neurons. The human CNS was long considered as incapable of neural regeneration. Approximately two decades ago, the fact that neurons regenerate continuously throughout life was recognized and gradually became widely accepted (Eriksson et al., 1998). The process of generating new fate-specified, functional neurons from neural progenitor cells, which are functionally incorporated into a neural circuit, is defined as neurogenesis (Ming and Song, 2005). Across different species, neural regeneration mainly takes place at the DG of the hippocampus and the SVZ along the lateral ventricle (Kuhn et al., 1996; Alvarez-Buylla and Garcia-Verdugo, 2002). Notably, the DG, which plays a crucial role in memory formation processes [e.g., pattern separation (Leutgeb et al., 2007)], is related to early memory loss in AD (Ohm, 2007).

Neurogenesis decline accompanies normal aging (Klempin and Kempermann, 2007). Patients with neurodegenerative disorders continuously lose neurons while neurogenesis is insufficient. For AD, accumulating evidence suggests that impaired neurogenesis plays a role in its pathogenesis (Hollands et al., 2016). Multiple molecules involved in AD pathogenesis [such as ApoE (Yang et al., 2011), PS1 (Gadadhar et al., 2011), and APP (Ghosal et al., 2010)] were recognized to take part in neurogenesis modulation. Conversely, inhibition of NSCs results in deterioration of cognitive processes, such as hippocampal-dependent memory (Imayoshi et al., 2008). Therefore, understanding the mechanism of neurogenesis dysfunction and intervening with neurogenesis represents an alternative AD therapeutic strategy. Neurotrophic factors and transcription factors involved in signaling pathways, the vascular and immune systems, metabolic factors, and epigenetic regulation are recognized to participate in regulating neurogenesis (Horgusluoglu et al., 2017).

Generally, neurogenesis can be modulated by multiple factors that are related to lifestyle, including learning (Gould et al., 1999), exercise (van Praag et al., 1999), social interaction (Stranahan et al., 2006), caloric restriction (Bondolfi et al., 2004), blood oxygen level (Lange et al., 2016), and even microbial colonization (Ogbonnaya et al., 2015). In this regard, advocating a healthy lifestyle exerts at least a mild effect on preventing or controlling AD in the long run. Future animal and epidemiological studies need to elucidate the best feasible individualized strategy for lifestyle modification combination that takes a patient’s genetic background into account. Apart from lifestyle modification, which exerts mild effects, several pioneering studies identified key molecules or drugs that rescue or reverse NSC dysfunction in elderly animal models.

### Proneurogenic Effects of Medications Available in the Clinic

Several drugs that are extensively used in the clinic have demonstrated robust proneurogenic effects in animals. Erythropoietin (EPO) is a cytokine that promotes hematopoiesis. Clinically, recombinant EPO is indicated for anemia. Its non-hematopoietic functions are being explored. During midgestation, EPO receptors are localized to regions of the neural tube that are responsible for neurogenesis (Alnaeeli et al., 2012). In the adult mouse brain, EPO receptors were detected primarily in the hippocampus, capsula interna, cortex, and midbrain (Digicaylioglu et al., 1995). Therefore, enhancing EPO receptor expression in the brain and increasing EPO is a potential strategy to enhance neurogenesis. In healthy young mice, 3 weeks of EPO administration significantly elevated the number of pyramidal neurons and oligodendrocytes (Hassouna et al., 2016). Furthermore, in an intracerebroventricular-streptozotocin rat model of sporadic AD, 2 weeks of EPO administration successfully prevented the memory deficit and the hippocampal neuronal loss induced by streptozotocin (Cevik et al., 2017).

Similarly, granulocyte colony-stimulating factor, a hematopoietic growth factor that stimulates proliferation and differentiation of neutrophil precursors, has been linked to enhanced neurogenesis (Schneider et al., 2005; Jung et al., 2006). In animal models of AD, restored memory was also observed (Tsai et al., 2007). If proven to be effective and safe in patients with AD, administration of these hematopoietic growth factors might be alternative options to modify symptoms.

Additionally, antidepressant drugs (primarily selective 5-HT reuptake inhibitors) have been established to play a crucial role in upregulating neurogenesis and achieving satisfying treatment response in patients with depression (Taupin, 2006; Hanson et al., 2011). Future studies need to determine whether antidepressants are efficacious for symptom reduction in patients with AD without comorbid depression. Further, additional research is needed to understand the relative contribution of enhanced neurogenesis and serotonin signaling, because previous studies have attributed reduced Aβ level and plaque formation in an aged APP/PS1 mouse model after EPO treatment to serotonin signaling regulation (Cirrito et al., 2011; Sheline et al., 2014).

### Correcting Aberrant Metabolism to Modulate Neurogenesis

Lipids are a major component of the brain. Aberrant lipid metabolism is highly associated with AD (Di Paolo and Kim, 2011), yet the exact mechanism remains to be fully uncovered. Recent evidence indicates its role in neurogenesis defect. Lipid droplets selectively accumulated in the SVZ were found to distort NSCs and their daughter neuroblasts before amyloid accumulation in a 2-month-old 3xTg AD mouse model (Hamilton et al., 2015). Using an imaging mass spectrometry-based lipidomics strategy to identify the lipid responsible, oleic acid accumulation within the SVZ was observed (Hamilton et al., 2015). Microanalysis of microdissected SVZs demonstrated local aberrant lipid metabolism in the 3xTg brain, including increased expression of stearoyl-CoA-desaturase, the rate-limiting enzyme of oleic acid synthesis. Strikingly, when oleic acid synthesis was inhibited by intracerebroventricular infusion with a stearoyl-CoA-desaturase inhibitor, NSC proliferation reduction in the DG and SVZ was rescued in 2-month-old 3xTg mice (Hamilton et al., 2015). Current knowledge on the interaction between lipid metabolism and NSCs in both physiological and pathological conditions is inadequate. With advances in cutting-edge technology to trace lipid metabolism in the brain, future research needs to unravel the roles played by diverse lipid metabolites and metabolic processes in NSC dysfunction and AD.

Mature neurons are highly dependent on the mitochondrial electron transport chain and oxidative phosphorylation to meet the high energy demand. Using pharmacological and genetic approaches, the metabolic switch from glycolysis to mitochondrial electron transport chain and oxidative phosphorylation was reported to be required for NSCs to give rise to intermediate progenitor cells in adult neurogenesis (Beckervordersandforth et al., 2017). Moreover, eliminating the function of mitochondrial transcription factor A (*Tfam*) replicated age-related neurogenesis decline in young mice. The most exciting part of this study is that short-term treatment with piracetam, a drug that improves mitochondrial function through a number of pathways, was sufficient to double the number of proliferating cells in an aged animal model. Numerous lines of evidence have indicated the involvement of mitochondrial dysfunction in the pathogenesis of AD (Swerdlow et al., 2014); therefore, additional studies are needed to analyze the role of NSC’s metabolic switch in AD pathogenesis.

Recent studies revealed that NSC behavior is regulated by multiple metabolism-related processes, such as oxygen consumption, ATP production, and reactive oxygen species signaling (Almeida and Vieira, 2017). The metabolic drug metformin (Wang et al., 2012; Fatt et al., 2015) was recognized to take part in neurogenesis modulation, suggesting that the complex nature of metabolism and neurogenesis remains to be fully investigated.

### Young Blood: ‘Resetting the Aging Clock’

Heterochronic parabiosis is an experimental method whereby the circulatory systems of young and elderly animals are joined and shared. Though controversial, there have been studies on the relationship between heterochronic parabiosis and rejuvenation for over a century (Conboy et al., 2013). The rationale behind these attempts is that there are signals from both the CNS itself and the body system outside the CNS that instruct neurogenesis in an age-related pattern. As NSCs in the CNS are exposed to blood vessels and cerebrospinal fluid, it is believed that by interfering with the blood carrying these signals, age-related neurodegeneration might be rescued. Administration of young plasma improves synaptic plasticity in the DG and elevated the cognitive function in elderly mice with the involvement of the cyclic AMP response element binding protein (Creb) signaling pathway (Villeda et al., 2014). Hunting for youth-promoting factors has attracted much research interest. Growth and differentiation factor 11 (GDF11) is one of the candidates that have shown promising potential regarding skeletal muscle, heart, and CNS rejuvenation (Jamaiyar et al., 2017). After systematic GDF11 administration, NSC proliferation in the SVZ was significantly elevated (Katsimpardi et al., 2014).

Conversely, chemokine CCL11, major histocompatibility complex component β2-microglobulin (β2-M), and transforming growth factor β (TGF-β) have been recognized as aging-promoting factors, which are elevated in the blood of the elderly and contribute to decreased neurogenesis and learning and memory (Villeda et al., 2011; Smith et al., 2015; Yousef et al., 2015). Furthermore, neurogenesis and cognitive functions can be mitigated in aged mice by reducing β2-M expression (Smith et al., 2015). In addition, inhibition of TGF-β1 signaling enhanced neurogenesis, as well as normalizing the β2-M level (Yousef et al., 2015).

It should be noted that enhancing neurogenesis does not equate to rejuvenating the brain. Rather than neurogenesis, elevated synaptic plasticity and hippocampal-dependent cognition were observed after intravenous administration of human cord plasma in aged mice (Castellano et al., 2017). Stem cell dysfunction is only one of the nine hallmarks of aging (López-Otín et al., 2013); therefore, future studies need to decipher the relative contribution of various modulators.

Shared mechanisms [e.g., synaptic plasticity (Hatanpää et al., 1999)] do exist between healthy aging and AD conditions; however, whether the interventions described above can sufficiently rescue cognitive decline in AD animal models and patients remains to be explored. The first clinical trial on transfusion of plasma from young donors to aged patients with AD is ongoing (identifier NCT02256306 on ClinicalTrials.gov). As previous studies have attributed better cognitive test results after plasma exchange to enhanced peripheral clearance of Aβ (Boada et al., 2009; Liu et al., 2015), plasma exchange studies need to use multiple outcome measures to evaluate neurogenesis elevation and increased amyloid clearance. Extra caution must be taken in clinical practice because of complications of plasma exchange (e.g., anaphylactic reaction). As more youth-promoting and aging-promoting factors are likely to be identified, it might be a prudent strategy to administer cocktail therapy comprising key modulators with known side effects.

### Looking Forward

A large number of current neurogenesis studies based on animal models are not fully applicable to humans. However, postmortem studies are limited by their retrospective study design, tissue damage after death, and incomplete patient history information. Although attempts have been made to identify key metabolic biomarkers (Manganas et al., 2007; Spalding et al., 2013), current non-invasive macroscopic neuroimaging measures in the brain, such as magnetic resonance imaging and positron emission tomography are not sufficiently specific, precise, or sensitive to detect neurogenesis (for a review, see Ho et al., 2013). Collaboration between different academic domains is urgently needed to develop precise *in vivo* neurogenesis detection techniques.

Limited by the available research techniques, current understanding of neurogenesis dysfunction in AD is lacking. Numerous studies have pointed out that neurogenesis alterations start relatively early in the course of AD progression (Mu and Gage, 2011; Unger et al., 2016), making it a promising target for early intervention or prophylaxis. Therefore, a mechanistic insight into when and how early neurogenesis alteration is triggered is required. Furthermore, with advances in neurogenesis detection techniques in adult humans, neurogenesis alteration might be an early marker of AD. By contrast, during AD progression, how the classic pathology (e.g., amyloid plaques) interacts with neurogenesis is not well understood. Interventions that promote the function of newly generated neurons in the context of AD pathology remains to be investigated.

To summarize, accumulating evidence suggests the promising potential of intervening with endogenous NSC dysfunction and deteriorated neurogenesis to improve AD-related cognitive decline. More molecules involved in neurogenesis are likely to exist, and identifying these molecules and their underlying mechanisms might pave the way for novel AD therapeutics.

## Transplanting Stem Cells to Support Neurons

Transplanting exogenous stem cells into CNS is an alternative strategy that has attracted much research interest. Significant effort has been made to engraft stem cells into degenerated neural tissue. However, the number of stem cells transplanted into brain decreased over time (Khoo et al., 2011). The efficacy of stem cell transdifferentiation into grafted tissue is low (Phinney and Prockop, 2007).

Accumulating evidence suggests that stem cells exert neurotrophic effects after transplantation (Lu et al., 2003; Martino and Pluchino, 2006). Transplanted stem cells elevate the levels of various factors, including BDNF (Blurton-Jones et al., 2009), glial cell line-derived neurotrophic factor (GDNF) (Kim S. et al., 2012), insulin-like growth factor 1 (IGF-1), Glucagon-like peptide-1 (GLP-1) (Klinge et al., 2011), vascular endothelial growth factor (VEGF) (Garcia et al., 2014), to exert a paracrine effect. Recent research comprising transplanting stem cells in animal models are listed in **Table 1**. Stem cells have been recognized to improve various cellular functions in animal models of AD, including synaptic strength (Blurton-Jones et al., 2009), neurogenesis (Kim S. et al., 2012; Kim D.H. et al., 2015), microglial activity (Lee et al., 2009a; Yang et al., 2013), angiogenesis (Garcia et al., 2014), mitochondrial function (Zhang et al., 2015), autophagy (Shin et al., 2014), and apoptosis (Lee et al., 2010). Stem cell transplantation influences AD via multiple mechanisms; therefore, it is promising compared with conventional treatments that target a single pathology.

**Table 1:** Stem cell transplantation studies on animal models of Alzheimer’s disease.

Reference	AD model	Stem cell source	Transplantation route	Observation time after transplantation	Molecular change	Cognitive change	Key findings
Blurton-Jones et al., 2009	3xTg-AD mice, 18-month-old	NSCs from mice with the same haplotype.	Stereotactically delivered to hippocampus.	1 months	Aβ, tau → BDNF ↑ Hippocampal synaptic density↑	Spatial learning↑ Novel object recognition↑	NSCs enhance cognition via BDNF without ameliorating Aβ or tau, as confirmed by loss-of-function study.
Lee et al., 2009a	Aβ injection to DG of C57BL/6 mice,	Bone marrow-derived MSCs from mice	Inject to bilateral hippocampus	7 and 30 days	After 7 days: Microglial activated to reduce Aβ Microglial morphology change After 30 days: No significant difference	NA	Microglial activation after MSC transplantation is involved in ameliorating Aβ.
Lee et al., 2009b Lee H.J. et al., 2012	2xTg-AD mice, 7 months 1 week old	Bone marrow-derived MSCs	Inject to bilateral hippocampus	6 weeks	Aβ, tau, β-secretase -1↓ Alternatively activated microglial activation↑	Spatial memory↑	Transplantation of MSCs ameliorate Aβ via microglial activation. Microglial phenotype switch from classic to alternative phenotype.
Ryu et al., 2009	Aβ injection to rat hippocampus	NPCs from rat embryos	Stereotactically delivered to hippocampus	7 days	NPCs tend to migrate to Aβ microgliosis↓ astrogliosis→ TNF-α↓ Attenuate Aβ-induced neuron loss	NA	Transplantation of NPCs attenuate Aβ-induced inflammation.
Lee et al., 2010	Aβ injection to DG of C57BL/6 mice, 12-week-old	Human umbilical cord-derived MSCs	Inject to bilateral hippocampus	7 days	Apoptosis in hippocampus↓ Oxidative stress↓ Glial activation↓	Spatial memory↑	Transplantation reduces Aβ-induced apoptosis in hippocampus.


Klinge et al., 2011	2xTg-AD mice, 3-month-old	Human bone marrow derived naive MSCs or MSCs transfected with GLP-1	Stereotactically injected to right ventricle	2 months	Without GLP-1 transfection: Aβ↓ With GLP-1 transfection: Aβ→ Glial and microglial suppression	NA	Encapsulated MSCs transfected with GLP-1 may cause unexpected microenvironment alteration, mechanism unknown.
Kim J.-Y. et al., 2012	2xTg-AD mice, 10-month-old	Human umbilical cord-derived MSCs	Inject to bilateral hippocampus, or cisterna magna	10, 20, and 40 days	Aβ in remote cortices (hypothalamus, amygdale, striatum) ↓ Neprilysin expression in microgli↑a Soluble intracellular adhesion molecule-1 (sICAM-1)↑	NA	Transplanted MSCs actively migrates to Aβ. sICAM-1 secreted by MSCs induces neprilysin expression in microglia via sICAM-1/LFA-1 pathway.
Kim S. et al., 2012	Tg2576 mice, 11-month-old	Autologous adipose derived stem cell	Intravenously injected or intracerebral injection	4 months	Aβ, APP↓ VEGF, GDNF, NT3↑ IL-10, anti-inflammatory cytokine↑ Endogenous neurogenesis↑	Spatial learning↑	Intravenous administration of adipose derived stem cells are permeable to blood–brain barrier in AD patients, representing a promising preventive strategy for AD.
Yang et al., 2013	2xTg-AD mice, 6-month-old	Neuron-like cell induced by D609 from human umbilical cord derived MSCs	Stereotactically injected to hippocampus	3 weeks	Aβ↓ M2-like microglial? Synapsin I↑ M2-like microgli↑a Proinflammatory cytokine↓ Anti-inflammatory cytokine↑	Spatial learning↑ Spatial memory↑	Transplantation of neuron-like cells differentiated from mesenchymal stem cell activates M2-like microglia to decrease Aβ and improve memory.
Blurton-Jones et al., 2014	3xTg-AD mice and Thy1-APP mice	Genetically modified NSCs stably secreting neprilysin.	Stereotactically delivered to subiculum (3xTg-AD mice), hippocampus (Thy1-APP mice).	3 months	Aβ↓ synaptic density↑ Aβ loads decreased not only in the surrounding area of exogenous NSC transplantation, but also in the projected areas.	NA	Utilizing the migratory NSC to deliver drug. Genetically modified NSC is an effective combinatorial therapy for AD.
Garcia et al., 2014	2xTg-AD mice, 6-, 9-, and 12-month-old	Bone marrow derived MSCs from mice, transfected with Vascular endothelial growth factor (VEGF)	Stereotactically injected to lateral ventrical	40 days	Neovascularization in hippocampus Aβ↓ in DG Astrocyte and microglial cell expression↓	Social recognition memory↑ Interest in novelty↑	MSC transplantation transfected with VEGF promotes neovascularization even in elder mice.
Ager et al., 2015	CaM/TetDTA mice, 7-month-old or 3xTg-AD mice, 19-month-old	Human NSC from donated fetal brain tissue	Stereotactically injected to hippocampus	4 weeks	Aβ, tau → Synaptic density↑	Spatial learning↑ Novel object recognition↑	Human NSCs migrate and differentiate into neurons and glia, elevate endogenous synaptogenesis.
Danielyan et al., 2014	2xTg-AD mice, 13-month-old	Bone marrow derived MSCs, macrophages and microglia	Intranasal delivery	2 weeks	Cells delivered to Olfactory Bulb, hippocampus, cortex and cerebellum. Delivered cells were Aβ positive.	NA	Intranasal stem cell delivery to CNS is a promising alternative route to avoid invasiveness.
Kim D.H. et al., 2015	2xTg-AD mice	Human umbilical cord blood derived MSCs, GDF-15 recombinant treatment	Repeated cisterna magna injections	12 weeks	Aβ↓ Synaptic vesicle↑ Endogenous neurogenesis in DG↑ GDF-15↑	NA	Repeated magna injections of MSCs is more beneficial than single injection. It enhanced neurogenesis and synaptic activity, modulated by paracrine effect of GDF-15.
Kim J.A. et al., 2015	Tg2576 mice 12-months-old and 15- months-old	NSCs from mouse embryo	Stereotactically injected to bilateral DG of hippocampus and the third ventricle	2 months	12-months-old: Aβ↓ inflammatory microglia activation↓ Neurogenesis↑ Synapse formation↑ 15- months-old: Aβ→	12-months-old: Spatial memory↑ 15-months-old: Spatial memory→	Early transplantation reduces neuropathology and rescues cognitive decline while transplantation for advanced stage is ineffective.
Zhang et al., 2015	2xTg-AD mice, 12-month-old	NSCs from mouse embryo.	Stereotactically injected to bilateral ventricles	5 and 10 weeks	Mitochondrial biogenesis related factors (PGC-1α, NRF-1, and COXIVP)↑ 10 months after transplantation, mtDNA in transplanted 2xTg-AD mice is equivalent to Wt mice. Mitochondrial fission/fusion balance alteration	Spatial learning and memory↑	NSC transplantation increases mitochondrial biogenesis by modulating the balance between mitochondria fission and fusion.
Misra et al., 2016	Intracerebroventricular -isoproterenol-induced rat	Bone marrow derived MSCs in combination with solid lipid nanoparticle (SLN) encapsulated galantamine hydrobromide (GH)	Intravenous delivery of stem cell, oral delivery of GH-SLNs	26 days	Antioxidant↑ Neurotrophic factor↑ Anti-apoptotic protein↑ Inflammatory mediators↓	Spatial memory↑	SLN encapsulated GH restores antioxidant levels in brain, enhancing the efficacy of stem cell treatment.
Wu et al., 2016	Tg 2576 mice, 16-month-old	BDNF overexpressing NSCs derived from mice	Stereotactically injected to hippocampus	2, 4, and 8 weeks	BDNF overexpression increases viability and neuronal fate of engrafted NSCs. Hippocampal BDNF and synaptic density↑	Spatial memory↑ Novel object recognition↑	Transplanting genetically altered NSCs is a promising strategy.

*↑ stands for elevated levels were observed*.

*↓ stands for decreased levels were observed*.

*→ stands for no significant change of levels were observed*.

*NA stands for not analyzed*.

*2xTg-AD mice express *APP* and *PS1* mutation*.

*3xTg-AD mice express *APP*, *PS1* and *MAPT* mutation*.

*5XFAD mice overexpress 3 *APP* mutations and 2 *PS1* mutations*.

*CaM/TetDTA is a mouse model characterized by loss of hippocampal CA1 neurons*.

### Transplanting Genetically Altered Neural Stem Cells

Most NSC transplantation studies successfully rescued cognitive dysfunction in animal models of AD, yet failed to ameliorate Aβ deposition (Blurton-Jones et al., 2009; Zhang et al., 2014; Ager et al., 2015). To maximize the efficacy of transplantation, a strategy that harnesses NSCs to deliver key disease-modulating proteins has been proposed. Overexpression of neprilysin, the key Aβ degrading enzyme, in transplanted MSCs significantly reduces synaptic loss and the Aβ level (Blurton-Jones et al., 2014). Other cellular functions that are of significant importance to AD are modulated by transfecting NSCs with other factors. BDNF-overexpressing NSCs induced a better recovery of the hippocampal BDNF level, synaptic density and stronger cognitive function (Wu et al., 2016). IGF-1 is another trophic factor that promotes differentiation toward neuronal cells and is essential for neural proliferation and survival (Russo et al., 2005). An *in vitro* study overexpressing of IGF-1 in cortical neurons demonstrated increased GABAergic neuron differentiation, increased VEGF production, and elevated survival of the transplanted cells (McGinley et al., 2016). Despite showing promising potential, this strategy faces major challenges. The safety and efficacy of transplanting genetically altered cells in humans has not yet been validated. Furthermore, this strategy requires stem cell genome alteration, which could face stricter regulatory restrictions in clinical translation.

### Inflammatory Responses Modulated by Mesenchymal Stem Cell Transplantation

Apart from NSCs, the most widely used source of stem cells for transplantation are MSCs. MSCs are cells that reside around blood vessels in bone marrow, supporting hematopoiesis and cartilage regeneration, and complementing the differentiated osteoblasts and adipocytes (Bianco et al., 2013). Not only do they differentiate into adipocytes, myocytes, osteoblasts, chondrocytes, and cardiovascular, and neurogenic cell types, but also tend to reside at sites of injury and inflammation (Karp and Leng Teo, 2009). Studies have confirmed modulation of inflammation after MSC transplantation.

Inflammation plays a critical role in AD pathogenesis (Heppner et al., 2015). Inflammatory responses in the CNS reflect endogenous efforts to clear pathological deposits. Microglia are the resident immune cells in the brain, which are involved in both neural protection and death. A number of studies have confirmed that MSC transplantation modulates microglial activity in the CNS to ameliorate Aβ (Lee et al., 2009a; Lee H.J. et al., 2012). Moreover, there are two opposite microglial phenotypes in the CNS: M1 and M2. M1 microglia releases pro-inflammatory cytokines such as TNF-α, IL-1β, and reactive oxygen species. M2 microglia, however, are anti-inflammatory. M2 microglia are induced by IL-4, IL-13, apoptotic cells, or other anti-inflammatory cytokines (Tang and Le, 2016). Several previous trials on mice confirmed that M2 microglia are involved in ameliorating Aβ after transplantation (Lee et al., 2009b; Ma et al., 2013; Yang et al., 2013). In this regard, targeting the M1/M2 microglia balance is a potential strategy to ameliorate inflammation in AD. CCL5 secreted by transplanted MSCs, for instance, has been recognized to activate M2 microglia (Lee J.K. et al., 2012).

The active homing mechanism of MSCs makes their systemic administration (e.g., intravenous injection) possible, which would possibly avoid direct invasion of the brain. Besides, they are convenient to access, lack ethical concerns, and have low immunogenicity. MSCs hold significant potential in clinical use. However, our current understanding of the MSC trafficking mechanism is lacking (Karp and Leng Teo, 2009). One recent study reported a low efficacy of MSC homing to lesion sites in an aged AD mouse model (Fabian et al., 2017). With increasing insights into the homing mechanism, manipulation to enhance the efficacy of transplanted MSCs that home specifically to the brain might benefit MSC transplantation.

### Extracellular Vesicles Derived From Stem Cells

Besides secreting soluble molecules such as BDNF (the classic paracrine effect), recent studies have explored the therapeutic potential of stem cell-derived extracellular vesicles (Katsuda et al., 2013a). Exosomes are cell-derived membrane vesicles containing lipids, proteins, mRNAs, and microRNAs. Recently, they have been recognized to be one of the key mediators of cell-to-cell communication. In addition to functional proteins, they transfer genetic information to recipient cells to regulate physiological or pathological processes (Valadi et al., 2007; Record et al., 2011). For instance, one study confirmed that injection of exosomes secreted by self-derived dendritic cells achieved 60% mRNA and protein knockdown of β-secretase 1 and 55% Aβ reduction using short interfering RNAs in wild-type mice (Alvarez-Erviti et al., 2011). From this perspective, it is likely that stem cells transmit tissue repair or regeneration signals to lesions via exosomes. Current research has revealed the potential of stem cell-derived exosomes in the treatment of stroke (Xin et al., 2013), myocardial ischemia (Lai et al., 2010), and liver fibrosis (Li et al., 2012). For AD, one recent study suggested that adipose tissue-derived MSCs secret exosomes that contain enzymatically active neprilysin when co-cultured with Aβ (Katsuda et al., 2013b). Looking forward, harnessing stem cells to either deliver designed drugs or secret a combination of molecules and RNAs that represent the body’s response to the pathological microenvironment with spatial precision is a promising strategy (El Andaloussi et al., 2013). Furthermore, although there is a long way to go, administration of exosomes derived from stem cells represents an alternative therapy for AD to circumvent relatively unsafe cell transplantation.

### Clinical Translation

There has been growing interest in exploring the potential of treating patients with AD using stem cell transplantation. Ongoing clinical trials intended to transplant stem cells into patients with AD are listed in **Table 2**. Various sources of MSCs, including human umbilical cord blood, placental tissue, autologous adipose tissue, and ischemia-tolerant MSCs, are being tested in clinical trials. Accessibility, invasiveness, potential tetratomic induction, proliferation rate, cost, and efficacy should be thoroughly evaluated and compared. The following paragraphs summarize several concerns and advances regarding transformation from the bench to the bedside.

**Table 2:** Selected clinical trials registered at ClinTrials.gov on stem cell therapy for Alzheimer’s disease as of October, 2017.

NCT number	Trial title	Interventions in experimental arm	Sponsor	Status
NCT02833792	A Phase IIa Study of Allogeneic Human Mesenchymal Stem Cells in Subjects With Mild to Moderate Dementia Due to Alzheimer’s Disease	Human adult ischemia-tolerant mesenchymal stem cells and lactated Riunger’s solution via intravenous administration	Stemedica Cell Technologies, Inc., United States	Recruiting starts from June, 2016
NCT02600130	A Phase I, Prospective, Randomized, Double-Blinded, Placebo-controlled Trial to Evaluate the Safety and Potential Efficacy of Longeveron Allogeneic Human Mesenchymal Stem Cell (LMSCs) Infusion Versus Placebo in Patients With Alzheimer’s Disease	Longeveron mesenchymal stem cells (high-dose or low-dose) via peripheral intravenous infusion	Longeveron LLC, United States	Recruiting starts from August, 2016
NCT02054208	A Double-Blind, Single-Center, Phase 1/2a Clinical Trial to Evaluate the Safety and Exploratory Efficacy of Intraventricular Administrations of NEUROSTEM Versus Placebo Via an Ommaya Reservoir in Patients With Alzheimer’s Disease	NEUROSTEM^®^ (human umbilical cord blood-derived mesenchymal stem cells) via intraventricular administrations	Medipost Co. Ltd., South Korea	Recruiting starts from February, 2014
NCT01297218	A Phase 1/2, Randomized, Double-Blind, Placebo-Controlled Study to Evaluate the Safety and Efficacy of AstroStem, Autologous Adipose Tissue Derived Mesenchymal Stem Cells, in Patients With Alzheimer’s Disease	Autologous adipose tissue derived mesenchymal stem cells via intravenous injection	Nature Cell Co. Ltd., South Korea	Recruiting starts from April, 2017
NCT02899091	A Randomized, Double-Blind, Placebo-Controlled, Phase I/IIa Clinical Trial for Evaluation of Safety and Potential Therapeutic Effect After Transplantation of CB-AC-02 in Patients With Alzheimer’s Disease	CB-AC-02 (placenta-derived mesenchymal stem cells) via injection	CHABiotech CO., Ltd., South Korea	Not yet recruiting
NCT02912169	An Open-label, Non-randomized, Multi-Center Study to Assess the Safety and Effects of Autologous Adipose-Derived Stromal Vascular Fraction (AD-SVF) Cells Delivered Intravenous (IV) and Intranasal in Patients With Alzheimer’s Disease	Autologous Adipose-Derived Stromal Vascular Fraction (AD-SVF) Cells Delivered Intravenous (IV) and Intranasal	Ageless Regenerative Institute, United States	Recruiting starts from November, 2015
NCT03297177	Use of Autologous Stem Cell Use in Neurological Non-neoplastic Disorders and Disease	Autologous stem/stromal cells derived from subdermal fat deposit via intravenous parenteral route	Healeon Medical Inc., United States	Recruiting starts from December, 2017

First, the efficacious time frame for AD treatment is not unknown. Multiple studies demonstrated increased synaptic strength in animal models after NSC transplantation (Bae et al., 2007; Blurton-Jones et al., 2009; Kim D.H. et al., 2015). Previous studies have pointed out that synaptic dysfunction occurs before plaque formation (Selkoe, 2002), and loss of synapses in the neocortex and hippocampus is the predominant factor that correlates with cognitive impairment in AD (Terry et al., 1991). Although not carefully tested in clinical trials, it is intriguing to think that NSC transplantation might protect patients with AD at an early stage. By contrast, AD is a progressive chronic disease that typically lasts several years after initial diagnosis; therefore, the appropriate time window for stem cell transplantation in the course of AD progression requires further exploration. A large number of preclinical studies used mouse models at a relatively young age or at an early-stage of disease progression, yet the observation time was not long enough. One study using the Tg2576 mouse model, which develops age-related cognitive defects, demonstrated that transplantation recovered cognition and ameliorated neuropathology in 12-month-old mouse, while transplantation failed to recover either cognition or neuropathology in a 15-month-old mouse (Kim J.A. et al., 2015). Future research needs to elucidate whether stem cell transplantation is efficacious for patients with AD in an advanced stage, and whether stem cell transplantation is efficacious and necessary for prophylactic purposes.

As transplantation research transforms from the laboratory to the clinic, large-scale stem cell transplantation requires proper quality control protocol. Recent preclinical studies on AD (Marsh et al., 2017) and cervical spinal cord injury (Anderson et al., 2017) demonstrated that clinical-grade stem cell transplantation might not be as effective as research-grade cell transplantation. To prioritize the efficacy and safety of transplantation for human patients, longer-term observation on multiple animal models after transplantation and more comparability tests on large-scale stem cell manufacturing are needed.

Invasion of the brain might be a major concern for elderly and weak patients with AD. Several studies reported novel methods to circumvent invasive surgery. Intranasal and intravenous routes are being explored (Kim S. et al., 2012; Danielyan et al., 2014; Kanamaru et al., 2015). Recent advances in brain imaging allow magnetic resonance imaging-guided focused ultrasound to target specific structures, involving transient disruption of the blood–brain barrier to deliver therapeutic stem cells from blood to the parenchyma (Burgess et al., 2011). Novel sources of stem cell are also being tested. For instance, dental pulp cells are cranial neural crest-derived multipotent cells that present neurotrophic properties (Mead et al., 2017). They are being tested as a potential stem cell source for transplantation in an AD model (Apel et al., 2009; Ahmed Nel et al., 2016).

Another concern in current clinical practice is transplantation rejection. To lower the risk of a serious immune response, researchers are exploring the potential of either autogenic stem cells (e.g., adipose tissue-derived MSCs or bone marrow-derived MSCs) or allogenic cells with hypo-immunogenic properties [e.g., umbilical cord-derived MSCs (Weiss et al., 2008)]. Alternatively, to help the engrafted cells avoid possible immune rejection, cell encapsulation techniques have been applied in several studies. With a polymeric semi-permeable membrane that allows the exchange of essential factors for cell metabolism, the encapsulated cells are protected from immune attack for long-term stable delivery of therapeutic agents. Several studies have used encapsulated somatic cells to deliver various growth factors to treat AD in animal models (Garcia et al., 2010; Spuch et al., 2010) and humans (Eriksdotter-Jönhagen et al., 2012; Wahlberg et al., 2012). One study demonstrated suppression of microglia and astrocytes using encapsulated MSCs transfected with GLP-1 (Klinge et al., 2011).

Last but not least, as has been described previously, the number of transplanted stem cell is prone to decrease over time. Increasing the survival rate of transplanted stem cells and lowering negative responses in the body after their death is crucial for sustaining a long-term therapeutic effect. These concerns need to be resolved before stem cell transplantation goes into clinical practice. Regulation and oversight should be strengthened to ensure that the tremendous potential of stem cells is fully realized.

### Investigating Alzheimer’S Disease by Reprogramming Techniques

It is difficult to obtain tissue samples from the human CNS to model disease; therefore, previous insights into AD relied heavily on post-mortem autopsy, which represents the pathology at the end of the disease, or in transgenic mice expressing or overexpressing *APP* or *PS* mutations. These animal models were developed on the basis of the prevailing amyloid cascade hypothesis, which holds that it is the deposition of APP cleavage products that causes the pathological changes. However, they are not capable of replicating the full spectrum of AD pathology observed in human patients, such as tau pathology, mutations in non-coding regions of the genome, and neurodegeneration. Furthermore, approximately 3–32% of patients clinically diagnosed with AD are amyloid-negative on positron emission tomography imaging (Ossenkoppele et al., 2015). The huge heterogeneity in AD patients requires them to be divided into subgroups or to be considered as individuals in terms of mechanistic studies and drug screening. The lack of proper disease models might be one of the reasons why drugs proven to efficiently ameliorate Aβ in animal models do not perform well in the human brain where much more complex pathologies are involved.

Induced pluripotent stem cells (iPSCs) are created using a technique that reprograms somatic cells back to the pluripotent-state by the overexpression of key transcription factors (Takahashi et al., 2007). The introduction of iPSCs has revolutionized neurological disease modeling. In 2011, the first AD model using iPSCs was reported (Yagi et al., 2011). Pluripotent stem cells were induced using five transcription factors (OCT4, SOX2, KLF4, LIN28, and NANOG) from fibroblasts of patients with familial AD. These iPSCs were then induced into neurons, which demonstrated typical pathology. Huge heterogeneity exists in patients with AD; therefore, the iPSC technique offers unique opportunities to study patients by subgroups and screen drugs in a patient-specific manner. The following paragraphs will review the phenotypes and drug reactions presented in recent iPSC-based AD models generated from a variety of patients.

### Familial Alzheimer’s Disease

Familial AD (fAD) affects about 0.5% of all patients with AD, the majority of which are autosomal dominant with full penetrance that typically presents before 65 years old. To generate amyloid-β peptides, two sequential cleavages of APP occur, cleavage by β-secretase in the extracellular space, and then by γ-secretase within the membrane. Mutations in *PS1*, *PS2*, and *APP* genes are the major causes of fAD.

The presenilin protein is an essential component of γ-secretase. Gamma-secretase cleaves at multiple sites; therefore, Aβ varies in amino acid length (36–43 residues). Aβ40 is the most abundant type and Aβ42 is most prone to self-aggregation (Haass and Selkoe, 2007). iPSC-based studies observed elevated Aβ42/Aβ40 (Yagi et al., 2011; Koch et al., 2012; Sproul et al., 2014). Furthermore, a γ-secretase inhibitor effectively reduced Aβ secretion (Yagi et al., 2011; Koch et al., 2012). NSCs from iPSCs with the PS1 L166P mutation were generated (Koch et al., 2012). That study demonstrated selectively decreased Aβ40 secretion and an elevated Aβ42/Aβ40 ratio. The Aβ42 level did not differ from the control; therefore, the authors concluded that partial dysfunction of γ-secretase occurs in the PS1 L166P mutation, while other γ-secretase functions remain intact. Sproul et al. (2014) studied neural progenitor cells derived from iPSCs carrying the PS1 A246E or M146L mutations. Compared with the control, molecular profiling identified 14 genes with altered expression in the *PS1* mutation lines. Among them, five genes were differentially expressed in late-onset AD. This study shed light on identifying genetic expression alterations, which will facilitate further studies on fAD pathogenesis.

Dissatisfying clinical outcomes cast doubt on the amyloid cascade hypothesis; therefore, its validity should be thoroughly tested using various models. Cells from patients with fAD carrying *APP* mutations provide models to study the relationship between Aβ and tau. Israel et al. (2012) studied two iPSC lines generated from patients with fAD who carried a duplication of the *APP* gene. Elevated Aβ40, active GSK3β (the kinase that phosphorylates tau at Thr231), phosphorylated tau at Thr231 and total tau was observed. To test if there was a direct causative relationship between the APP processing product and phosphorylated tau and active GSK3β, β-secretase and γ-secretase inhibitors were added. Notably, only the β-secretase inhibitor treatment partially reduced phosphorylated tau and active GSK3β levels, indicating that APP processing (products other than Aβ) is responsible for tau Thr231 phosphorylation. This study also confirmed the assumption that early endosomes are present in iPSC-iNs, implying that these early endosomes take part in modulating APP processing.

Muratore et al. (2014) explored the relationship between APP processing and tau in the APP V717I mutation. The APP V717I mutation alters the initial cleavage site of γ-secretase, causing altered APP cleavage by both β-secretase and γ-secretase. The iNs demonstrated increased levels of both Aβ42 and Aβ38, and increased total and phosphorylated tau. Early Aβ antibody treatment reverses tau, suggesting a partially causal relationship between altered APP processing (Aβ) and tau formation.

Moore et al. (2015) studied the relationship between APP processing and tau in different patients with AD of different genetic backgrounds. iPSC lines which altered the APP dosage (*APP* duplication) or ε-cleavage site (APP V717I) demonstrated elevated total or phosphorylated tau levels, while *PS1* mutants (Y115C and intron 4), which elevated the Aβ42/Aβ40 ratio, did not. Furthermore, a β-secretase inhibitor, which prevents the β-C-terminal fragment (CTF) from forming, reduced the intracellular tau level. Meanwhile, a γ-secretase inhibitor that promotes β-CTF aggregation elevated the intracellular tau level. Therefore, Moore et al. (2015) proposed an intriguing hypothesis that the APP cleavage product (β-CTF) is involved in regulating tau pathology. Whether or not β-CTF is related to advanced tau/NFT pathology remains to be investigated.

Kondo et al. (2013) studied the phenotype of the APP-E693Δ mutation, a rare autosomal recessive mutation, using iPSCs. Intriguingly, rather than extracellular Aβ aggregation, intracellular accumulation of Aβ oligomers was observed in this APP-E693Δ line. In addition, intracellular Aβ aggregation leads to a cellular stress response causing endoplasmic reticulum and oxidative stress. One of two sporadic patient lines demonstrated the same phenotype as APP-E693Δ mutation, while the APP-V717L mutation did not. Furthermore, only the lines with intracellular Aβ are responsive to Docosahexaenoic acid treatment. The authors proposed dividing patients with AD into the intracellular Aβ type and extracellular Aβ type to achieve personalized treatment.

### Sporadic Alzheimer’s Disease

The majority of patients with AD suffer from sporadic AD (sAD). As revealed by several iPSC studies, large heterogeneity exists among the phenotypes and drug responsiveness of patients with sAD (Israel et al., 2012; Kondo et al., 2013). Large-scale genome-wide association studies have identified numerous susceptible genetic variations in patients with late-onset sAD (Lambert et al., 2013), demonstrating the complex genetic nature of sAD. However, the biological functions of those key genes associated with the pathogenesis of AD have not been well studied. Furthermore, altered levels of Aβ were not observed in a large proportion of patients with sAD (Toledo et al., 2013), and tau predicts dementia symptoms better than Aβ (Brier et al., 2016). Thus, conventional animal models, which do not involve tau pathology, are not appropriate to study sAD. Several recent studies using iPSC-based modeling gained insights into the function of these genes and provided examples of how to study sporadic diseases.

Apolipoprotein E (*APOE*), the gene encoding the key protein for lipid catabolism has been recognized to play a key role in the pathogenesis of sAD. Sortilin-related receptor, L [DLR class] (SORL1) is a neuronal APOE receptor that is predominantly expressed in the CNS. Young et al. (2015) reported the function of *SORL1* single nucleotide polymorphisms in non-coding regions in an iPSC-based model. When treated with BDNF, protective phenotypes (carrying one or two protective alleles) responded with significantly higher *SORL1* expression and Aβ reduction compared with those of the risk phenotypes (carrying two risk alleles). Furthermore, *SORL1* knockdown confirmed that BDNF-induced Aβ reduction is dependent on SORL1 expression. This study indicated that neurotrophic factors such as BDNF are potentially regulated by disease risk-related genetic mutations. Furthermore, this study implied the necessity of detailed stratification of patients with sAD to tackle the pathogenetic mechanisms.

Hossini et al. (2015) studied genetic expression by transcriptome analysis and demonstrated the first protein interaction network from one patient with sAD. By analyzing the transcriptome, the authors reported the upregulation and downregulation of several genes that differed from fAD-associated genes. Moreover, the authors observed ubiquitin-proteasome system dysfunction in this patient with sAD compared with an age-matched control. Protein interaction network analysis revealed the involvement of APP and GSK3β.

### Three-Dimensional Modeling and Chimeric Modeling

In humans, hyperphosphorylated tau aggregates in dendrites and axons to form dystrophic neurites, and aggregates in cell bodies to form NFTs. As shown in **Table 3**, conventional iPSC-based modeling did not demonstrate robust NFTs. Choi et al. (2014) developed a three-dimensional human NSC-derived *in vitro* model of AD. Matrigel containing extracellular matrix proteins was used as the support. By limiting Aβ diffusion, this three-dimensional culture promoted Aβ aggregation and its downstream cascade. In addition, it closely mimics the real cell environment with regard to vertical cell growth, the synaptic distance between cells, and cell maturation (D’Avanzo et al., 2015). In Choi et al.’s study, iPSC lines carrying *APP* and *PS1* mutations in three-dimensional culture successfully demonstrated robust extracellular Aβ plaques and NFTs. Furthermore, β-secretase and γ-secretase inhibitors reduced the Aβ level as well as the tau level, supporting the amyloid cascade hypothesis.

**Table 3:** iPSC modeled Alzheimer’s disease.

Reference	Disease and mutation	Tested cells (induced from iPSC)	Phenotype reported	Effective drug treatment
Yagi et al., 2011	fAD: *PS1* A246E mutation, *PS2* N141I mutation	Neurons	Elevated Aβ42/Aβ40.	Compound E (γ-secretase inhibitor) and compound W (Aβ42 inhibitor) reduced Aβ42 and Aβ40. High dose compound W reduced Aβ42/Aβ40 ratio.
Israel et al., 2012	fAD: duplication of *APP* sAD	Neurons	Aβ(1-40), phospho-tau, aGSK-3β increase, large RAB5-positive early endosomes.	β-secretase inhibitor reduces phospho-tau and aGSK-3β.
Koch et al., 2012	fAD: *PS1* L166P mutation, *PS1* D385N mutation	iPSC and embryonic derived NSCs	Elevated Aβ42/Aβ40. Decreased Aβ40, while Aβ42 did not differ from control. Partial loss of γ-secretase function.	γ-secretase inhibitor and non-steroidal anti-inflammatory drugs reduce Aβ.
Duan et al., 2014	sAD: ApoE3/E4	Basal forebrain cholinergic neurons	Elevated Aβ42/Aβ40. Elevated sensitivity to calcium influx and glutamate toxicity.	Low dose of γ-secretase inhibitor elevates Aβ secretion in sAD, while it typically reduces Aβ in fAD.
Kondo et al., 2013	fAD: *APP*-E693Δ mutation and *APP*-V717L mutationsAD	Neurons	Intracellular Aβ aggregation in APP-E693Δ line, while no significant extracellular plaque aggregation was observed. Endoplasmic reticulum and oxidative stress response in APP-E693Δ line.	Docosahexaenoic acid (DHA) reduces stress response in *APP*-E693Δ and one of two sporadic patients.
Muratore et al., 2014	fAD: *APP*-V717L mutation	Neurons	Elevated Aβ42 and Aβ38. Increased β-secretase cleavage of APP. Altered γ-secretase cleavage site Hyperphosphorylated tau.	Aβ-specific antibody reduce elevated tau.
Sproul et al., 2014	fAD: *PS1* A246E mutation, *PS1* M146L mutation	Neural progenitor cells, neurons	Elevated Aβ42/Aβ40, more apparent in NPCs than neurons. 14 genes (e.g., *NLRP2*, *ASB9*, *NDP*) are recognized to alter expression in *PS1* mutated patients’ NPCs, 5 of them involved in late-onset AD as well.	
Hossini et al., 2015	sAD	Neurons	Elevated GSK3β activity and phosphorylated tau. Generate an AD-related protein network. Ubiquitin-proteasome system function is down-regulated in sAD.	γ-secretase inhibitor down regulates phosphorylated tau.
Moore et al., 2015	fAD: *PS1* Y115C mutation, *PS1* intron 4 mutation, *APP* V717I mutation, *APP* duplication	Neurons	All lines demonstrated increased Aβ42 generation. *APP* mutations show elevated phenotype while *PS1* mutations did not.	γ-secretase inhibitor increased intracellular tau, β-secretase inhibitor reduced intracellular tau. γ-secretase modulation reduced intracellular tau.
Young et al., 2015	sAD: SORL1 SNPs (risk variants and Protective variants)	Neurons		BDNF treatment reduces Aβ by *SORL1*–dependent upregulation in protective homozygotes or heterozygotes. In risk homozygotes, BDNF treatment is not effective in reducing Aβ. Overexpression of *SORL1* cDNA ameliorates Aβ.
Woodruff et al., 2016	fAD: *PS1* ΔE9 mutation, *APP* V717F mutation, *APP* Swedish mutation	Neurons	APP and LDL endocytosis and soma-to-axon transcytosis of lipoproteins dysfunction in fAD.	β-secretase inhibitor recovers endocytosis function.
Jones et al., 2017	fAD and sAD	Astrocytes Neural progenitor cells	Atrophy and abnormal localization of astrocytes from both fAD and sAD, while NPCs did not show evident pathology.	

Another type of three-dimensional culture system, termed organoids or spheroids, is a scaffold-free self-organizing structure (Lancaster et al., 2013). Raja et al. (2016) developed brain organoids from iPSCs derived from patients with fAD. The organoid successfully recapitulated Aβ aggregation, hyperphosphorylated tau, and endosome abnormalities. Treatment with β-secretase and γ-secretase inhibitors reduced the Aβ and tau pathology. Lee et al. (2016) generated a three-dimensional neuro-spheroid culture from blood cell-derived iPSCs from patients with sAD. Similar to Raja et al.’s findings, the three-dimensional neuro-spheroids demonstrated Aβ aggregation, and hyperphosphorylated tau. Notably, the authors reported reduced Aβ-ameliorating efficacy of the β-secretase and γ-secretase inhibitors in three-dimensional model compared with a two-dimensional model. More research is needed to determine the cause of the diminished drug efficacy in the three-dimensional model. This type of three-dimensional modeling offers a great opportunity to study the role of cellular interactions (e.g., astrocytes and microglia involvement and neural cytoskeletal malfunction) in AD progression.

Chimeric modeling is another strategy to mimic the complex nature of multiple cellular interactions in the human brain. Espuny-Camacho et al. (2017) reported grafting healthy human neural precursor cells derived from iPSCs into the frontal cortices of immunodeficient newborn transgenic *APP/PS1*-21 mice. When exposed to Aβ, microglia, and astrocytes, the engrafted healthy human neurons developed significant degeneration, as observed by decreased synaptic density and dystrophic neurites, which has not been fully recapitulated in previous animal models. In comparison, the transplanted mouse cells did not develop striking neurodegeneration, indicating that it is crucial to use human neurons rather than murine neurons. Six months after transplantation, there was substantial human cell loss. Furthermore, the transplanted human cells demonstrated necrosis, 3R to 4R tau expression switching, and hyperphosphorylated tau accumulation. Intriguingly, the transplanted neurons died in the absence of NFT, leaving future studies to understand mechanism that generates NFTs and the cause of neuronal death. This novel chimeric model demonstrated promising potential to study human-specific AD pathogenesis.

### Mimicking Aging in Reprogramming

Interestingly, aging is a major risk factor for AD (Lindsay et al., 2002). However, the iPSC technique resets age-associated traits (e.g., cellular senescence, telomere shortening, and mitochondrial dysfunction) back to a fetal stage (Mahmoudi and Brunet, 2012), which potentially contribute to AD pathogenesis (Moreira et al., 2010). This might lower the validity of iPSC modeling. Stress exposure (e.g., free radicals, hypoxemia), triggering progeria syndrome pathways has been suggested to accelerate aging in iPSCs (Vera and Studer, 2015; Soria-Valles and López-Otín, 2016).

Apart from resetting somatic cells to a primitive pluripotent state, the reprogramming technique allows iNs to be directly generated from somatic cells, without an embryonic state. Activating the transcription factor combination BAM (Brn2, Asc11, and Myt11) using exogenous ectopic expression of a transgene has been proven to be sufficient to generate functional iNs from mouse fibroblasts (Vierbuchen et al., 2010). With the addition of factor NeuroD1, human fibroblasts can be transdifferentiated into functional mature neurons (Pang et al., 2011). The combination of a micro-RNA (miR-124) and two transcription factors (Myt11 and Brn2) achieved successful conversion of human fibroblasts into functional iNs (Ambasudhan et al., 2011). Non-viral reprogramming methods, which are less invasive to the genome, are being explored. Black et al. (2016) reported that endogenous BAM transcription factors expression could be engineered using the CRISPR/Cas9 system to convert fibroblasts into neuronal cells. Small molecule cocktails have also been reported to directly induce neurons. By adding a cocktail of seven small chemicals that regulate neuronal transcription factor expression, Hu et al. (2015) converted fibroblasts from patients with fAD carrying *APP* or *PS1* mutations into neuronal cells, and observed amyloid and tau phenotypes, showing the promising potential of using iNs to model AD. A large percentage of iNs induced by this cocktail are glutamatergic cells. Although iNs are considered to retain aging-related genetic and epigenetic characteristics that better mimic AD, the fate of iNs is not under precise control. Further work is needed to decipher the molecular mechanisms that govern the differentiation toward a specific neuronal subtype, for example, in AD, cholinergic neurons in the basal forebrain.

The paragraph above summarized several methods to convert fibroblasts into functional iNs *in vitro*, which might shed light on AD modeling and disease mechanism. Several studies have explored the therapeutic potential of converting glial cells to neural cells in injured brain tissue (Guo et al., 2014; Su et al., 2014). Guo et al. (2014) reported the successful reprogramming of reactive glial cells into functional neurons in an AD model. Overexpression of one transcription factor, NeuroD1, turned astrocytes into glutamatergic neurons, whereas it turned NG2 cells (oligodendrocyte precursor cells) into glutamatergic and GABAergic neurons. This study exploited the pathological gliosis that inhibits neural regeneration and survival in the AD brain as a source of direct reprogramming. Further work is needed to explore whether these neurons induced under pathological conditions are beneficial to improve pathological responses.

### Looking Forward

Tremendous variations in the genetic background and life experience (epigenetic change) exist in patient-derived iPSC lines. Current advances in genome editing techniques (e.g., CRISPR/Cas9, zinc finger nuclease, helper-dependent adenovirus) allow us to observe different phenotypes caused by mutations of interest, while keeping the genetic background constant (Liu et al., 2011; Soldner et al., 2011; Kwart et al., 2017). For instance, Fong et al. (2013) exploited genetically engineered iPSCs to study the tauopathy phenotype. Using a zinc finger nuclease, the authors created isogenic iPSC lines carry wild-type *TAU*, and heterozygous or homozygous *TAU*-A152T mutation. Mutation dose-dependent neurodegeneration and axonal degeneration were observed.

Efforts have been made to transplant cells derived from iPSCs. Apart from the human-mice chimeric model (Espuny-Camacho et al., 2017), human iPSC-derived dopaminergic neurons were transplanted into a primate Parkinson’s disease model (Kikuchi et al., 2017). In Kikuchi et al.’s study, high grafted cell survival rate and no tumor were observed in 2 years, demonstrating the bright future of transplanting iPSC-based cells in terms of low tumorigenicity and high survival rate. Mandai et al. (2017) reported the first case of transplanting a retina derived from autologous iPSCs into a patient with age-related macular degeneration. Creating iPSCs for each patient is time-consuming, laborious, and expensive; therefore, efforts have been made to establish iPSC banks that are presumed to be sufficient to find at least partial HLA matched donor cells in Japan, the United States, and Europe. The first clinical trial of allogenic iPSC transplantation has been initiated by the same group in Japan to transplant retinas into patients with age-related macular degeneration. AD typically occurs in the elderly, and autologous cells are prone to contain accumulated genetic abnormalities that are potentially harmful; therefore, transplanting cells derived from HLA-matched iPSCs is a strategy worthy of further exploration.

## Conclusion

Alzheimer’s disease is a complex neurodegenerative disorder that involves multiple cell types and a large variety of cellular activities. Identifying key molecules involved in the modulation of endogenous neurogenesis and intervening with them might be a preliminary, but promising, strategy to prevent or even reverse AD. Although several pioneering studies have demonstrated elevated neurogenesis in terms of metabolism and plasma exchange in animal models, future studies need to test the efficacy of these manipulations in human patients.

**FIGURE 1:**
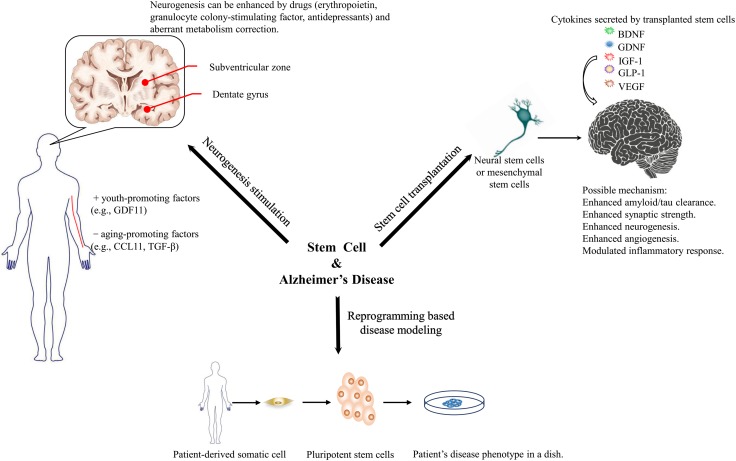
Three aspects that stem cell technology might benefit Alzheimer’s disease research and therapeutics.

Transplanting stem cells to substitute for lost neurons is another intuitively feasible strategy. However, studies have confirmed that the main benefit of stem cell transplantation is a neurosecretory effect. Various neurotrophic factors involved in modulating multiple cellular functions that promote the amelioration of pathological features and cognition in animal models have been recognized. There has been increasing commercial interest to transform current advances in transplantation into clinical practice on human patients. Various stem cell sources and transplantation routes are being studied to promote the efficacy and safety of transplantation. Regulatory rules from governments should catch up with the growing enthusiasm for stem cell transplantation (see the summaries in **Figure 1**).

One of the major hurdles in developing therapeutics for AD and studying its pathogenesis is the lack of animal models that fully recapitulate the pathological features observed in humans. iPSCs have revolutionized AD modeling because they make it possible to generate neuronal cells directly from patients. A substantial amount of research has proven their potency in modeling diseases and screening drugs. Long-term controversies over the disease mechanism (e.g., the challenged amyloid hypothesis) can be tested in iPSC-based models. Three-dimensional modeling and chimeric modeling have been proposed because they aggregate amyloid potency or/and better mimic various cellular interactions that take place in the patient’s brain. Direct reprogramming techniques circumvent the intermediate embryonic state; thus, aging-related features that potentially contribute to AD pathogenesis are maintained. Genome editing techniques allow isogenic comparison of various mutations while keeping the genetic background constant. The phenotypes and drug reactions of different iPSC lines from various patients have been accumulating; therefore, future research might develop detailed patient stratification rules to provide patients with personalized drug regimens. Combined with high-throughput drug screening, future translational studies will be easier.

## Author Contributions

YF gathered the initial case information and drafted the initial manuscript. TG participated in the case gathering and drew the figure. BZ reviewed and revised the manuscript. JP reviewed and revised the manuscript and supervised the gathering of case information. All authors approved the manuscript as submitted and agreed to be accountable for all aspects of the work.

## Conflict of Interest Statement

The authors declare that the research was conducted in the absence of any commercial or financial relationships that could be construed as a potential conflict of interest.

## Abbreviations

Aβ: amyloid beta
AD: Alzheimer’s disease
ApoE: apolipoprotein E
APP: amyloid precursor protein
BDNF: brain-derived neurotrophic factor
CNS: central nervous system
DG: dentate gyrus
fAD: familial Alzheimer’s Disease
iNs: induced neurons
iPSC: induced pluripotent stem cell
MSC: mesenchymal stem cell
NFTs: neurofibrillary tangles
NSC: neural stem cell
PS1: presenilin 1
PS2: presenilin 2
SVZ: subventricular zone.
